# Foregrounding motivational climate in university physical education: a mixed-methods latent profile analysis with classroom prescriptions

**DOI:** 10.3389/fspor.2026.1748720

**Published:** 2026-02-02

**Authors:** Chunmei Li, Yuling Li, Chengyun Wu, Richard Peter Bailey, Nadia Samsudin

**Affiliations:** 1 Faculty of Social Sciences and Liberal Arts, UCSI University, Kuala Lumpur, Malaysia; 2 School of Physical Education and Music, Qilu University of Technology, Jinan, China; 3 School of Music and Dance, Shandong Yingcai University, Jinan, China

**Keywords:** empowering motivational climate, self-efficacy development, latent profile analysis, physical education pedagogy, formative feedback and tired tasks

## Abstract

**Background:**

Evidence on empowering motivational climates in Chinese university physical education is limited, particularly regarding how students cluster into distinct perception patterns and how such heterogeneity can be converted into profile-specific teaching actions that support competence and self-regulation. This study identified latent profiles of university students’ physical education perceptions and derived actionable implications aligned with empowering climates.

**Methods:**

In a mixed-methods study, undergraduates from 10 universities in Shandong Province (China) completed a survey used to identify latent perception profiles across six learning-related dimensions and to test demographic predictors. Student and teacher interviews were thematically analyzed to interpret profile mechanisms and inform recommendations.

**Results:**

Among 1,259 students, three clearly separated profiles emerged (entropy = 0.892). Females had higher odds of membership in lower-support profiles [largest OR = 1.732, 95% CI (1.199, 2.504)]. Second-year students were more likely than first years to belong to the moderate-to-higher profile relative to the low profile [OR = 1.483, 95% CI (1.071, 2.054)]. Interviews suggested that performance visibility and threat constrained engagement, whereas progress referenced assessment with explanatory feedback strengthened perceived competence and self-regulation, helping explain profile differences.

**Conclusion:**

Findings position motivational climate as a key lever for improving Chinese university PE and provide a profile-informed implementation pathway. We recommend a classroom prescription combining tiered tasks, structured choice, and low-threat, progress-referenced formative assessment with explanatory feedback. Implementation should prioritize first-year classes and female students, consolidate through self-monitoring and peer assessment, and scale via structured autonomy and resource optimization.

## 1 Introduction

In Chinese higher education, university physical education (PE) is expected to promote students’ physical activity (PA), mental health, and core competencies. It is also a comparatively malleable instructional setting, because teachers can adjust classroom organization, task design, and assessment cues more directly than in many academic subjects (1). These design features matter because they shape students’ immediate judgements about “can I learn this?” and “is it worth learning?”, which in turn influence participation quality and sustainability (2).

However, persistent tensions remain in university PE. When course goals are framed primarily in utilitarian terms (e.g., “pass the test” or “get the credit”), intrinsic motivation can be undermined and engagement becomes fragile (3). In addition, homogenized teaching routines reduce opportunities for tired and personalized support, especially in classes with wide skill variance (4). Assessment is often delayed or presented as a single, high-stakes judgement, which makes progress harder to see and weakens self-monitoring and self-efficacy (5). Finally, controlling instructional cues may suppress autonomy and enjoyment, discourage first attempts, and increase error avoidance (6). These issues are compounded when teachers and students diverge in their perceptions of value, difficulty, and success criteria, widening motivational gaps and creating a chronic mismatch between instructional organization and motivational support (2, 7).

To organize these concerns, this study foregrounds motivational climate as a central instructional construct. Here, an empowering climate refers to teaching that communicates informational goals, emphasizes learning processes, and supports students’ autonomy, competence, and relatedness (e.g., clear success criteria, process-focused feedback, and supportive peer organization) (8, 9). In contrast, controlling climate relies on external pressure, social comparison, and person-focused evaluation (e.g., public ranking, fixed-pace drills, and outcome-only grading), which can heighten threat and reduce willingness to try (10, 11).

Within an empowering motivational climate, autonomy support, task involvement, and social support are widely viewed as the most critical and actionable levers for instructional design (12). Autonomy support emphasizes providing students with meaningful, curriculum-aligned choices within a framework of clear instructional goals and classroom norms, such as task options aligned with lesson objectives, negotiable difficulty levels, and explicit, transparent assessment requirements (13, 14). This approach is well suited to Chinese university PE, where classes are often large, timetables and facility allocations are relatively fixed, and equipment and space must be shared among many students (4). In such contexts, autonomy cannot be treated as unbounded free choice. Instead, “structured autonomy” choice embedded within clear rules and consistent standards helps preserve classroom order and assessment consistency while offering progressive learning pathways for students with diverse foundations (15, 16).

These pedagogical cues align with two complementary theoretical accounts. Self-Determination Theory (SDT) explains motivational quality through autonomy, competence, and relatedness support; need-supportive teaching predicts higher engagement and more adaptive intentions (17, 18). Social Cognitive Theory (SCT) clarifies how modelling, progressive mastery, and self-reflection build self-efficacy and sustain self-regulation through goal setting and strategic control (19, 20). Together, SDT and SCT suggest that climate-relevant inputs (task design, choice, feedback, and assessment cues) should influence engagement and persistence by shaping perceived competence, threat, and efficacy.

Despite growing interest in motivational climates, evidence in university PE still leaves three core gaps. First, literature is dominated by variable-centered analyses, which describe average associations but seldom reveal whether students form distinct configurations of perceived support across behavioral, individual, and contextual dimensions (21, 22). Second, subgroup comparisons are typically reported as mean differences, without testing whether they reflect qualitatively different student types (profiles) with different instructional needs (23). Third, many studies stop at explanation and do not specify which concrete, modifiable classroom levels such as task tiering, structured choice routines, and progress-referenced assessment. Differentiate configurations and can be combined into implementable prescriptions to strengthen competence and self-regulation (24).

To address these gaps, we adopt a person-centered approach. Latent Profile Analysis (LPA) can identify common perception configurations and test how profile membership varies across background variables (25). However, profiling alone is insufficient for practice: profiles require interpretive evidence to specify mechanisms and actionable levers. Therefore, we integrate LPA with qualitative interviews to explain why profiles differ and how classroom cues shape participation quality and confidence.

Accordingly, this study aims to: (i) map undergraduates’ latent profiles of PE perceptions; (ii) examine profile distribution across key background variables; and (iii) elucidate classroom and organizational levers that differentiate profiles and can be converted into actionable prescriptions. Conceptually, the study makes heterogeneity explicit in university PE perceptions; methodologically, it provides a reusable measurement–profiling benchmark; and practically, it offers a profile-informed pathway for shifting from teaching-centered routines to learning-centered, precision-oriented reform in university PE.

## 2 Materials and methods

### 2.1 Participants and setting

Undergraduate students were recruited from ten universities in Shandong Province, China. Universities were randomly selected from the official Shandong Provincial Department of Education institutional list using a RAND-based procedure in Microsoft Excel. Of 1,400 questionnaires distributed, 1,300 were returned (92.86%). After prespecified screening (completion time, attention checks, and multivariate outliers), 41 questionnaires (3.15%) were excluded, leaving 1,259 valid responses. Participation was voluntary with written informed consent.

### 2.2 Measures and qualitative tools

#### 2.2.1 Quantitative measure

Students completed the University Students’ Perceptions of Physical Education Scale (US-PPE), developed for Chinese undergraduates (26) and grounded in Social Cognitive Theory. The US-PPE contains 20 items rated on a 7-point Likert scale, covering six domains: habitual behavior (HB; 3 items), self-efficacy (SE; 3 items), attitudes/experience (AE; 4 items), skills/knowledge (SK; 3 items), classroom climate (CC; 3 items), and facilities/equipment/norms (FEN; 4 items).

In the present sample, internal consistency was high (Cronbach's α = .967). Because very high α values can partly reflect scale length and overlapping content, we additionally inspected item total correlations and inter-item correlation patterns, to evaluate potential redundancy while retaining domain coverage.

#### 2.2.2 Interview guides

To complement the quantitative profile findings and explore classroom processes underlying students’ perceptions, we conducted semi-structured interviews with students and teachers. This format ensured consistent coverage of key topics while allowing flexible probing for concrete, experience-near accounts, balancing comparability and depth across participants (27, 28).

Guide development and grounding. Interview guides were developed using a structured, literature-informed process consistent with published methodological recommendations for semi-structured interview guide construction, including: (a) identifying prerequisites and study aims, (b) retrieving and integrating prior evidence, (c) drafting an initial guide, (d) pilot testing/refining prompts, and (e) finalizing the guide for transparent reporting (29). The guide content was mapped to the study's focal constructs (e.g., classroom organization, task differentiation, feedback practices) and to mechanisms highlighted in the qualitative results (e.g., efficacy-building feedback, self-regulation routines).

Tool quality/credibility indicators. As qualitative interviews are not evaluated via psychometric indices in the same way as questionnaires, we strengthened tool quality and credibility through: (a) content coverage and transparent guide-development procedures (29), (b) standardized administration of core topics across participants with flexible probing (27, 28), and (c) triangulation of student and teacher accounts with quantitative profile patterns (30). All interviews were conducted individually, audio-recorded with consent, and transcribed verbatim.

### 2.3 Procedure

Surveys were administered during regular PE sessions using standardized written instructions and an invigilated completion process across universities. To reduce social desirability and evaluation concerns, surveys were completed anonymously, and students were informed that participation (or non-participation) would not affect grades. Teachers were not involved in data handling.

Following quantitative profiling, we implemented a purposive qualitative phase to explain profile differences and to capture mechanisms that could account for profile membership. Student interview recruitment used a multistage purposive strategy: 8 students from each latent profile (C1/C2/C3) were selected with balanced gender and year representation (total = 24), and 6 instructors teaching different year groups were interviewed. All interviews lasted approximately 30–40 min. Purposeful sampling and multistage sampling are recommended in mixed-method designs when the goal is to obtain information-rich cases and to ensure coverage of key subgroups identified in the quantitative phase (31).

Interviews were conducted on campus in a private setting by trained interviewers using a standardized flow: (1) confidentiality and non-evaluative framing, (2) general course experiences, (3) critical-incident prompts targeting assessment, feedback, grouping and task differentiation, and (4) closing improvement suggestions. Semi-structured interviewing guidance informed the use of neutral prompts, relational rapport, and probe strategies to balance relationship and rigor (27, 28).

The qualitative sample size was planned to achieve thematic breadth across the three profiles and is consistent with empirically grounded guidance on interview numbers needed to reach code/meaning saturation in many focused qualitative studies (32). Integration of qualitative and quantitative evidence was supported through systematic comparison across data sources and presentation via joint display logic (31).

The study was registered on the Open Science Framework as an Open-Ended Registration (created on 1 Nov 2025; project: https://osf.io/evwd7). The registration documents are the study protocol, instruments/interview guides, inclusion/exclusion rules, and the planned quantitative (profile modelling and covariate tests) and qualitative analytic strategies.

### 2.4 Quantitative analysis

Descriptive statistics and reliability indices were computed in SPSS 27.0, and LPA was estimated in Mplus 8.3. Based on prior person-centered research in motivational and educational settings, we expected a small number of interpretable profiles (approximately three to four) representing low, moderate, and higher perceived support configurations, while allowing empirical fit and interpretability to determine the final solution. The optimal number of profiles was selected using both substantive and statistical criteria. Substantive criteria included theory coherence, clear qualitative distinctness between profiles, parsimony, and avoidance of very small classes (<5% of the sample) (33). Statistical criteria included log-likelihood and information criteria (AIC, CAIC, BIC, ABIC; lower values indicate better fit), likelihood ratio tests (BLRT; VLMR-LRT), and classification quality (entropy and average posterior probabilities) (34–36).

Demographic covariates were examined using the R3STEP approach with classification-error correction. Covariates included gender, grade, major, and region of origin, selected because these characteristics are consistently linked to differential learning opportunities, confidence, and participation experiences in university PE. Associations are reported as odds ratios (OR) with 95% confidence intervals. No missing data.

### 2.5 Qualitative analysis

Interview data were analyzed using reflexive thematic analysis following six phases (37). Two analysts independently coded an initial subset of transcripts and then held three consensus meetings to compare interpretations, resolve discrepancies through discussion and return to the data, and refine an evolving codebook. The final framework was applied to the full dataset, and themes were iteratively reviewed, defined, and named.

Sampling adequacy was assessed using thematic saturation/information power logic. Theme development stabilized by approximately the fifth interview round, with later interviews primarily elaborating existing themes. Reflexivity was supported through analytic memos and debriefs; given the analysts’ familiarity with the Chinese university PE context, assumptions were documented upfront, decisions were recorded in an audit trail, and peer challenge was used to test interpretations against the data.

### 2.6 Integration

A mixed-methods design integrated the quantitative and qualitative strands. LPA first identified distinct perception profiles, which guided purposive interview sampling by recruiting students from each profile. Semi-structured interviews then examined students’ experiences of classroom organization and teacher cues, focusing on task difficulty, assessment, feedback meanings, and opportunities for choice. Integration occurred at interpretation and recommendations: qualitative themes were used to explain between-profile differences and, where needed, to qualify quantitative patterns by showing that similar ratings could reflect different meanings. Joint displays aligned profiles with themes and illustrative quotes, and convergence, complementarity, and divergence were documented to inform a staged instructional prescription.

## 3 Results

### 3.1 Participant characteristics

A total of 1,259 valid cases were obtained (Table 1). The sample was predominantly female (female *n* = 947, 75.2%; male *n* = 312, 24.8%). Disciplinary background was broadly balanced (science *n* = 626, 49.7%; humanities *n* = 633, 50.3%), as was grade of study (first-year *n* = 605, 48.1%; second-year *n* = 654, 51.9%). Regarding place of origin, the majority were from rural areas (country *n* = 897, 71.2%; city *n* = 362, 28.8%). Overall, the sample was balanced by discipline and year, with some skew by gender and origin (higher proportions of females and rural students), providing a basis for subsequent stratified analyses or controlling these variables in multivariable models.

**Table 1:** Participant characteristics.

Valid		Frequency	Percent	Valid percent	Cumulative percent
Gender	Male	312	24.8	24.8	24.8
	Female	947	75.2	75.2	100.0
Subject	Science	626	49.7	49.7	49.7
	Humanities	633	50.3	50.3	100.0
Grade	First-year	605	48.1	48.1	48.1
	Second-year	654	51.9	51.9	100.0
Region	City	362	28.8	28.8	28.8
	Country	897	71.2	71.2	100.0

Based on 1,259 valid cases, descriptive statistics for the six dimensions are reported in Table 2. All means were above the scale midpoint (4 on a 1–7 scale), indicating that, on average, students tended to endorse (rather than remain neutral about) statements reflecting supportive PE experiences and learning-related resources. However, the means were not near the upper end of the scale, suggesting room for pedagogical improvement, particularly in self-efficacy and attitudes/experience, which were closest to the midpoint relative to other dimensions.

**Table 2:** Descriptive statistics.

Valid	*N*	Minimum	Maximum	Mean	Std. deviation
HB	1,259	1.00	7.00	5.4302	1.30371
SE	1,259	1.00	7.00	4.8681	1.33707
AE	1,259	1.00	7.00	5.0477	1.36098
SK	1,259	1.00	7.00	5.3985	1.29420
CC	1,259	1.00	7.00	5.7652	1.20903
FEN	1,259	1.00	7.00	5.8225	1.20382
Valid *N* (listwise)	1,259				

Importantly, standard deviations were relatively large (SD = 1.20–1.36) and observed scores spanned the full theoretical range (1–7) across all dimensions. This dispersion indicates that the overall “positive” mean co-existed with substantial between-student variability, consistent with the presence of distinct subpopulations. This pattern provided a strong empirical rationale for adopting a person-centered approach (LPA) to model heterogeneity that would be masked by variable-centered averages.

### 3.2 Latent profile analysis of students’ perceptions of physical education

**Figure 1:**
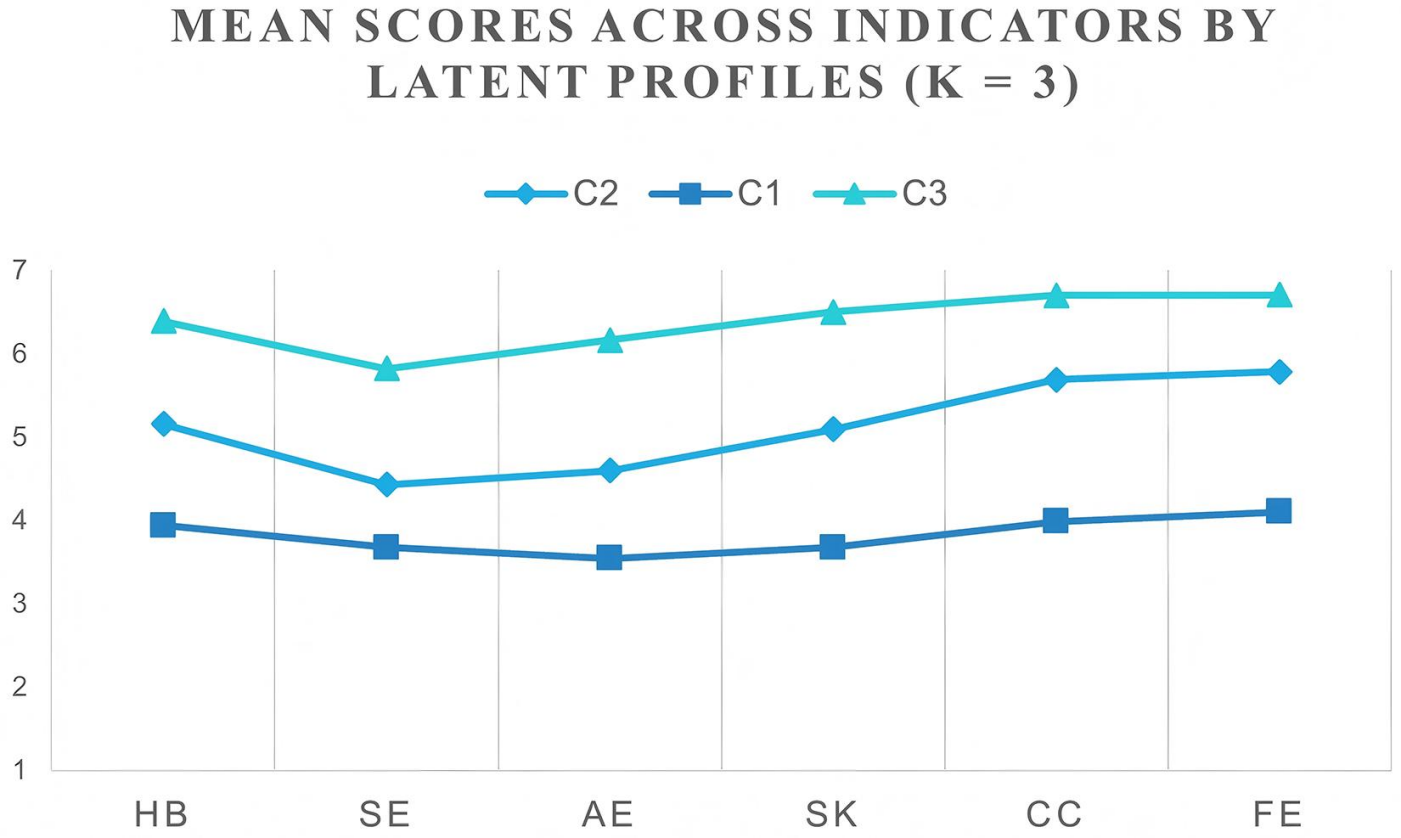
Latent 3-class model.

Comparing the 1–5 class solutions (Table 3), AIC/BIC/ABIC decreased monotonically with additional classes, suggesting improved fit with higher model complexity. However, inferential tests and interpretability were considered jointly. For the 2- and 3-class models, both the VLMR-LRT and BLRT were significant (both *p* < .001), indicating that the step from k−1 to k was warranted (Chen et al., 2024). The 3-class solution showed good classification quality (entropy = 0.890), with class proportions of 34.95%, 21.45% and 43.61% (*n* = 440/270/549), each of adequate size. Although the 4- and 5-class models further reduced information criteria (e.g., 4-class: AIC = 19,578.31; 5-class: AIC = 18,983.54), both yielded minimal classes (2.62% and 1.11%, respectively), undermining stability and interpretability, and their VLMR/BLRT results were only marginally significant (4-class: VLMR *p* = .021; 5-class: VLMR *p* = .021). Guided by lower information criteria, significant stepwise tests, sufficient classification quality, and adequate class sizes (33, 59), we selected the 3-class solution as the optimal and more parsimonious model. Figure 1 depicts the three-profile pattern of students’ PE perceptions.

**Table 3:** Fit indices of latent profile analysis on perceptions of physical education.

Model	LL	AIC	BIC	ABIC	Entropy	BLRT	VLMR	Class probability (%)	Number of profiles
						*p*	*p*		
1	−12,600.531	25,225.063	25,286.720	25,248.602					
2	−10,732.120	21,502.240	21,599.864	21,539.511	0.918	0.0000	0.0000	40.985, 59.015	516, 743
3	−10,121.575	20,295.150	20,428.740	20,346.152	0.890	0.0000	0.0000	34.948, 21.446, 43.606	440, 270, 549
4	−9,756.155	19,578.309	19,747.866	19,643.042	0.907	0.0223	0.0210	2.621, 20.572, 35.187, 41.620	33, 259, 443, 524
5	−9,451.767	18,983.535	19,189.058	19,061.999	0.914	0.0218	0.0207	1.112, 18.745, 34.392, 21.604, 24.146	14, 236, 433, 272, 304

Table 4 reports mean item responses on the six indicators across the three latent profiles. C3 (*n* = 549, 43.61%) scored highest on all six indicators, with an overall average across dimensions of 6.382 and indicator means ranging from 5.823 to 6.705; this profile is labelled high across all dimensions. C1 (*n* = 270, 21.45%) scored lowest on all indicators (overall average = 3.820; range = 3.544–4.101) and is labelled low across all dimensions. C2 (*n* = 440, 34.95%) fell between the other two (overall average = 5.125; range = 4.428–5.783) and is labelled moderately high across all dimensions. Classification accuracy was good, with mean posterior probabilities of 0.969 (C1), 0.940 (C2) and 0.950 (C3). The pattern shows “parallel separation”: C3 uniformly highest, C1 uniformly lowest, and C2 intermediate.

**Table 4:** Item response means and standard errors in latent profile analysis of physical education.

Class label	*n*	Percent (%)	Avg posterior prob. for own class	HB		SE		AE		SK		CC		FEN	
				Mean	SE	SE	SE	Mean	SE	Mean	SE	Mean	SE	Mean	SE
C1 low across all dimensions	270	21.45	0.969	3.94	0.076	3.674	0.069	3.544	0.066	3.674	0.061	3.987	0.068	4.101	0.08
C2 moderately high across all dimensions）	440	34.95	0.94	5.158	0.079	4.428	0.069	4.596	0.078	5.095	0.085	5.692	0.081	5.783	0.074
C3 high across all dimensions	549	43.61	0.95	6.392	0.063	5.823	0.091	6.164	0.078	6.502	0.045	6.704	0.025	6.705	0.025

### 3.3 Multinomial logistic regression

We used the R3STEP approach based on the three-class solution to examine whether demographic characteristics predicted profile membership while accounting for classification error. Covariates included gender, grade, major, and place of origin, selected because they represent common sources of variation in PE participation opportunities, confidence, and perceived support in Chinese university settings. Associations are reported as odds ratios (OR) with 95% confidence intervals (Table 5).

**Table 5:** R3STEP multinomial logistic regression predicting class membership.

Predictor (covariate)	Coding	Reference class	Comparison class	Logit b	SE	OR	CI Lower	CI Upper	*p*	sig
Gender	1 = Male, 2 = Female	C1	C3	−0.55	0.188	0.577	0.399	0.834	**0** **.** **003**	**
Gender	1 = Male, 2 = Female	C1	C2	−0.23	0.205	0.795	0.531	1.188	0.263	
Gender	1 = Male, 2 = Female	C3	C1	0.55	0.188	1.732	1.199	2.504	**0** **.** **003**	**
Gender	1 = Male, 2 = Female	C3	C2	0.32	0.164	1.377	0.998	1.9	0.052	
Subject	1 = Science, 2 = Humanities	C1	C3	−0.236	0.155	0.789	0.583	1.069	0.127	
Subject	1 = Science, 2 = Humanities	C1	C2	0.028	0.166	1.028	0.743	1.423	0.866	
Subject	1 = Science, 2 = Humanities	C3	C1	0.236	0.155	1.267	0.935	1.715	0.127	
Subject	1 = Science, 2 = Humanities	C3	C2	0.264	0.143	1.303	0.984	1.724	0.065	
Grade	1 = First-year, 2 = Second year	C1	C3	−0.159	0.155	0.853	0.63	1.155	0.303	
Grade	1 = First-year, 2 = Second year	C1	C2	0.394	0.166	1.483	1.071	2.054	**0** **.** **018**	*
Grade	1 = First-year, 2 = Second year	C3	C1	0.159	0.155	1.173	0.866	1.588	0.303	
Grade	1 = First-year, 2 = Second year	C3	C2	0.235	0.144	1.264	0.954	1.675	0.102	
Region	1 = City, 2 = Countryside	C1	C3	−0.204	0.174	0.816	0.58	1.147	0.242	
Region	1 = City, 2 = Countryside	C1	C2	−0.165	0.186	0.848	0.589	1.222	0.377	
Region	1 = City, 2 = Countryside	C3	C1	0.204	0.174	1.226	0.872	1.724	0.242	
Region	1 = City, 2 = Countryside	C3	C2	0.039	0.157	1.04	0.765	1.413	0.803	

Rows with *p* < .05 are bolded; OR and 95% CI are presented in separate columns.

* *p* < 0.05.

** *p* < 0.01.

Significant effects and practical relevance. Two effects were robust. First, compared with males, females had 73% higher odds of belonging to the low-support profile (C1) rather than the high-support profile (C3) [OR = 1.73, 95% CI (1.20, 2.50), *p* = .003], highlighting female students as a priority group for threat-reduction and competence-support strategies. Second, compared with first-year students, second-year students had 48% higher odds of belonging to the moderately high profile (C2) rather than the low profile (C1) [OR = 1.48, 95% CI (1.07, 2.05), *p* = .018], suggesting modest year-related improvement in perceived support that may still fall short of the high-support configuration. A further gender contrast (C2 vs. C3) was borderline (OR = 1.38, *p* = .052) and is interpreted cautiously as suggestive rather than conclusive evidence.

Non-significant effects and interactions. Academic major and place of origin showed no statistically reliable associations with profile membership (ORs approximately 0.79–1.30 with confidence intervals crossing 1), indicating that once other covariates were considered, profile membership was not strongly patterned by discipline or urban/rural origin in this sample. These null findings suggest that the key heterogeneity captured by the profiles is more closely tied to gender- and year-linked experiences than to broad disciplinary or origin categories, although small effects cannot be ruled out given the width of some confidence intervals. We did not include interaction terms in the primary R3STEP models to maintain parsimony and interpretability; future work could test targeted interactions to identify especially high-risk subgroups.

### 3.4 Qualitative data analysis

Drawing on triangulation between semi-structured student and teacher interviews and quantitative findings, we inductively identified five themes (with associated sub-themes) capturing the coupled relations among motivation, self-efficacy, self-regulation, classroom structure, and resource accessibility (Table 6). Cross-source comparison indicated strong convergence between the qualitative evidence and the LPA profiles and the key R3STEP results.

**Table 6:** Joint display linking LPA profiles (C1–C3) to qualitative themes and profile sensitive instructional levers.

Theme	Quantitative pattern	Qualitative evidence	Integrated inference	Practical levels
Motivation and self-efficacy	Females more likely to belong to C1.	Females reported worries about losing marks and looking foolish in graded or publicly visible tasks, reducing first attempts and challenge uptake.	Gendered threat perception and perceived cost of failure help explain why females concentrate in C1.	Low-threat, progress-referenced assessment; specific process feedback; reduce early public exposure; structured peer modelling to trigger C1 to C2.
Study habits and self-regulation	Second-year students are more likely to belong to C2.	C3 students maintained a goal practice review adjust loop and regular logging. C2 students practiced but reviewed less, making engagement easier to disrupt.	Grade effects likely reflect self-regulation routines. C2 to C3 progression depends on stabilizing monitoring, review, and adjustment.	Goal decomposition; practice logs; brief self and peer check-ins; guided review and adjustment, prioritizing first-years.
Classroom climate	Movement from C1 to C3.	Tiered goals plus flexible grouping created small attainable successes and increased C1 participation. High expectations plus positive tone was associated with C3. Negative emotional cues entrenched C1.	Profile differences are shaped by how tasks, grouping, and assessment signals influence perceived safety and belonging.	Use tiered tasks, flexible grouping, and progress referenced formative assessment with explanatory feedback. Avoid public ranking and outcome only grading.
Skills and knowledge scaffolding	C2 and C3 exceed C1 on skills and knowledge.	Stepwise demonstrations and salient cues shortened mastery time and increased confidence. Transfer of conditioning principles to extra class plans distinguished C3 from C2.	Scaffolding supports early mastery; transfer and load regulation underpin the C2 to C3 shift.	Stepwise modelling and error cues; explicit principles; simple out-of-class plans with recording and adjustment.
Facilities and resources	Direct effects are limited.	Magnifying organizational efficacy and learning returns.	Resources act as multipliers by enabling efficient organization and more practice-feedback cycles.	Optimize scheduling and station layout; low-equipment variants to increase active time and practice density.

Theme 1: Motivation and Self-efficacy Loop (linked to gender differences).

Outcome expectations and body-related anxiety. Many female students reported worries about ‘losing marks/looking foolish’ in graded or publicly visible tasks, tending to reduce first attempts and the level of challenge undertaken. This pattern aligns with the R3STEP finding that females were more likely to fall into C1, suggesting gendered threat perception as a risk factor for entry into the least advantageous profile. A minority counter-pattern was observed in small classes, emphasizing progress-referenced criteria, where female students’ avoidance markedly diminished.

Feedback visibility. When teachers provided immediate, specific, and actionable process feedback, stepwise guidance and concrete corrections, students rapidly developed a sense of control and early success; self-efficacy increased accordingly. This was a frequent trigger of movement from C1 to C2. By contrast, generic comments (e.g., “try harder,” “not standard,” etc.) yielded limited efficacy gains.

Peers and role models. Students starting from a lower baseline benefited most from proximal peer modelling and mutual assistance. Imitable, catch-up-able referents reduced failure expectancies, raised attempt frequency and consolidated the positive feedback loop. For higher-level students, peer roles were expressed more through demonstration quality and finer-grained assistive feedback.

Taken together, under the joint influence of negative outcome expectancies and body-related anxiety, students were more inclined to adopt avoidance strategies, curtailing first attempts and challenge willingness. Conversely, when teachers supplied visible, specific and practicable process feedback, supplemented by modelling and support from peers of comparable ability, students obtained clear corrective cues and immediate success experiences. Self-efficacy rose, which increased re-attempt intentions and actual engagement, manifesting as a transition from C1 to C2.

Theme 2: Study Habits and Self-regulation (explaining C3–C2 differences).

Goal decomposition and self-monitoring. Second-year students more frequently broke unit aims into staged sub-goals and combined self-assessment with peer assessment for process monitoring, consistent with the R3STEP result that second-year students were more likely to enter C2. A minority of first-year students also formed goal hierarchies under strong teacher guidance, but these were less stable.

Time management and consolidation. Regular practice, post-class consolidation, and fitness/homework logging co-occurred strongly with C3. A hallmark of C3 students was a stable “goal–practice–review–adjust” loop. C2 students typically had goals and practiced, but their reviewing and strategic compensation were insufficient, leaving their engagement more susceptible to disruption and fluctuation.

Students who translated unit aims into actionable, staged goals and engaged in process-focused self-assessment after each practice selected targeted strategies and difficulty levels. Subsequent reviews identified technical deviations and load mismatches, prompting iterative adjustment of the next stage's goals and plans. This closed loop transformed self-regulation from sporadic behavior into a stable disposition, sustaining purposeful engagement and continuous improvement amid contextual variability manifesting as a transition from C2 to C3.

Theme 3: Classroom Climate and Task Structure.

Tiered grouping and successful experiences. Using tiered goals alongside homogeneous/heterogeneous grouping reliably generates “small, attainable successes” and markedly increases C1 students’ willingness to participate. Where task difficulty is not tiring and performance is primarily rank-ordered, C1 students are more prone to marginalization.

Assessment friendliness. Formative assessment centered on progress-referenced criteria and explanatory feedback substantially reduces female students’ withdrawal tendencies and promotes sustained attempts. By contrast, high stakes, one-shot grading and public comparison amplify the perceived cost of failure and dampen participation.

Teacher expectations and emotional climate. High expectations coupled with a positive emotional tone are consistently associated with C3. When teachers use growth-oriented narratives to frame “difficulty” as a trainable challenge, students can better maintain effort and persistence. Negative emotional cues (impatience, sarcasm) are linked to entrenchment in C1.

Tiered tasks combined with progress-referenced, explanatory formative feedback increase the frequency of “small wins”, reduce threat, and raise engagement. Simultaneously, high expectations and a favorable affective climate reframe difficulty as trainable, strengthening perseverance and facilitating progression from C1 through C2 to C3 from passive participation to stable self-regulation.

Theme 4: Skills and Knowledge Scaffolding.

Visual modelling and stepwise instruction. Stepwise demonstrations, immediate correction of key errors, and salient cues markedly shorten the pathway to skill mastery and align with upward trends in quantitative skill. Without scaffolding, students tend to “train by feel”, with slower and poorer acquisition.

Cross-context transfer. Transferring health knowledge and principles of physical conditioning beyond class to everyday settings such as self-devised training plans with recording and adjustment is a major driver of progression from C2 to C3. Once students “know why and how”, they are more likely to sustain practice autonomously and optimize training loads.

In skill construction, visual modelling combined with stepwise scaffolds accelerates early mastery and builds confidence. Subsequently, applying principles of fitness and load regulation to extra-class practice (plan–execute–record–adjust) elevates understanding from procedural operation to conceptual internalization, catalyzing self-driven, sustained practice and strategy optimization manifesting as movement from C2 to C3.

Theme 5: Facilities and Accessibility, Limited Direct Effects, Amplifying Classroom Gains.

Regional differences: small in perception, crucial in scheduling. Consistent with the quantitative findings, perceived urban–rural differences were limited. However, the efficiency of equipment scheduling and spatial layout directly shaped the quality of students’ experiences, helping to explain the “marginal effects” of facilities, equipment and regulations on participation.

Refined Classroom Organization. Process optimization and simple-equipment strategies markedly compress waiting time and expand effective practice duration and density within a single lesson. As a result, existing pedagogical interventions are magnified: students encounter more frequent immediate feedback and “small wins”, steeping the slopes of engagement and skill acquisition. This mechanism shows how organizational redesign can yield substantial learning and participation gains without additional resources.

Profile differences were not driven by any single dimension but emerged from a multi-factor coupling of gendered threat perception, efficacy acquisition, self-regulatory capacity, classroom structure and resource accessibility. In Phase 1, low-threat, process-oriented feedback and proximal peer modelling should be used to break the ‘negative expectancy–avoidance’ loop, initiating a C1 → C2 cycle of attempt–feedback–success. In Phase 2, goal decomposition, practice logs, and systematic review consolidate a stable self-regulatory state underpinning C2 → C3 progression. Throughout, tiered tasks and assessment-friendly practices create early successes, while high teacher expectations and a positive affective tone reframe difficulty as a trainable challenge. Although not sufficient on their own, resources and equipment function as critical multipliers, magnifying organizational efficacy and learning returns.

## 4 Discussion

Drawing on quantitative and qualitative evidence, this study maps university students’ PE perceptions in Shandong and identifies three stable latent profiles. Mean scores on all six dimensions were above the midpoint, but FEN and CC were highest whereas SE and AE were comparatively lower. This pattern indicates a structural imbalance: structural/resource support is more visible than psychological/experiential support. R3STEP results showed two robust predictors. Females were more likely than males to belong to C1 rather than C3, and second-year students were more likely than first years to belong to C2 rather than C1. Major and place of origin showed no stable effects after adjustment. Interviews aligned with these patterns and pointed to linked mechanisms involving threat perception, efficacy building, self-regulation, classroom structuring, and access to resources.

This imbalance is not only classroom level; it is plausibly institutionally constructed. University PE is often delivered in large classes under fixed timetables and shared facilities. These conditions encourage investment in visible infrastructure and orderly organization, which is reflected in higher FEN and CC. By contrast, teacher preparation and in-service development may prioritize technique instruction, safety management, and syllabus compliance over climate-specific competencies such as low-threat task design, autonomy-supportive communication, and progress-referenced formative assessment. When monitoring and accountability relieve auditable indicators (e.g., attendance, standardized tests, uniform criteria), teachers are incentivized to use fixed pacing and one-off performance tests. Such routines are efficient, but they can leave students’ competence beliefs, enjoyment, and self-regulation under-supported (38).

Accordingly, higher FEN and CC do not automatically yield “willingness to attempt, persistence, and reflective review.” Lower SE and AE suggest that judgements of “Can I cope?” and “Do I enjoy this?” remain underdeveloped. This is consistent with evidence that low self-efficacy predicts self-handicapping and reduced PA investment (39). C1 students were especially constrained on SE/AE. Interview data highlighted anxiety about public performance, high-stakes assessments, and social comparison. These experiences imply that assessment is often perceived as judgement, rather than information for improvement. Autonomy support therefore becomes hinging: it links structural conditions to experience living by supporting needs and building efficacy (40).

The prominence of visibility-related threat is also locally plausible. In Chinese educational settings, social norms around avoiding public mistakes and maintaining “face” can increase the perceived cost of failure in performance-based PE tasks, particularly when demonstrations occur in front of peers and criteria are perceived as fixed. Under these norms, public comparison and high-stakes tests amplify the threat of “making a mistake and being seen.” This threat suppresses first attempts and reduces willingness to try challenging tasks (41). It offers a context-sensitive explanation for why psychological/experiential support may lag structural support even when facilities and organization are adequate.

Gender differences were educationally meaningful. Females’ higher likelihood of C1 membership (≈1.7 × males) is consistent with research showing higher social physique anxiety and fear of failure in socially evaluative and body-exposing contexts, including evidence from Chinese samples (42). Crucially, these gendered patterns are not purely individual. They can be amplified by institutionalized routines such as public ranking, uniform drills, and assessment designs that foreground display without sufficient scaffolding. Autonomy supportive teaching can reduce fear of failure and counter controlling cues (14). This enables a practical sequence: threat reduction → small-success accumulation → efficacy growth (43). It can be operationalized through low-risk demonstrations, stepwise error-correction, peer support, and tiered tasks, alongside progressive self-monitoring and reflection (40).

Second-years’ higher likelihood of C2 suggests gains in self-monitoring and strategic compensation with experience. Yet this may partly reflect acclimatization to institutional expectations rather than intentional climate support. Evidence indicates that autonomy-supportive teaching improves engagement and can spill over to out-of-class activity (44). Self-regulated learning practices, goal decomposition, process self-evaluation, and iterative review also support sustained practice and achievement, and can reduce dropout when embedded early (45, 46). Embedding these routines in first year may therefore shorten the C1 → C3 transition and shift students from passive completion to active self-regulation.

Passive and active learning, and the shift from teaching-centered to learning-centered practice. Taken together, the three profiles can be interpreted as a continuum from passive compliance to active, self-regulated learning. C1 reflects passive completion: students rely on teacher directions, suppress first attempts under public performance and high-stakes assessment cues, and rarely convert feedback into sustained practice. C2 represents guided participation: students engage when tasks are well structured and support is available, yet self-monitoring routines remain inconsistent. C3 reflects active learning: students report higher self-efficacy and enjoyment, set proximal goals, seek feedback, and practice beyond class. This breakdown clarifies why we frame structured autonomy as a practical route for shifting from teaching-centered delivery to learning-centered precision reform. Teachers retain clear goals, safety rules, transparent criteria, and pacing (structure), while including students through bounded choice, tiered task pathways, peer roles/modelling, and progress-referenced formative feedback that reframes assessment from judgement to information for improvement (43, 47). Autonomy is thus enacted within structure, aiming to move students from passive participation to active self-regulation without compromising order or safety (44, 48).

Within an outcome–mechanisms–prescriptions frame, we propose a two-stage pathway. For C1 → C2 (initiation), progress-referenced assessment with explanatory feedback can build efficacy and self-regulation (49). Tiered tasks and peer modelling provide attainable mastery and vicarious experiences (50, 51). Bounded choice and negotiated goals promote internalization while preserving structure (48). For C2 → C3 (consolidation), goal decomposition, practice logs, peer assessment, and systematic debriefs can institutionalize self-regulation (52, 53). Visualized demonstrations and stepwise scaffolding further reduce threat. A plan–do–record–adjust cycle supports transfer and maintenance (54, 55).

These prescriptions align with broader evidence that mastery climates relate to positive affect and satisfaction, whereas disempowering/performance climates relate to negative experiences (56). Empowering climates promote participation and persistence through need satisfaction and autonomous motivation (12, 57). Disempowering climates increase burnout and dropout intentions through need frustration and controlled motivation (10, 11). Autonomous motivation and competence satisfaction also predict long-term PA maintenance (58).

In sum, structural and psychological engines remain weakly coupled. The main bottleneck is the institutionally shaped mechanisms that build self-efficacy, positive experience, and self-regulation. Leveraging the large sample, the latent profiles offer a practical basis for learning-centered precision reform: rather than uniform changes for all students, instructional levels can be aligned with profile needs. For C1 (especially females and first-year students), priorities are threat reduction and early mastery through low-risk demonstrations, tiered tasks, and immediate process feedback to trigger first attempts and rebuild efficacy. For C2, the focus is to consolidate active learning by institutionalizing self-regulation routines (e.g., goal decomposition, practice logs, peer assessment, and structured debriefs) alongside autonomy-supportive communication. For C3, instruction should strengthen transfer by linking skills/knowledge to out-of-class practice planning and sustained PA. Overall, coupling visible infrastructure with climate- and learning-process-focused pedagogy is crucial to convert attendance-based participation into durable, self-regulated engagement. Future work should test causal effects and boundary conditions using longitudinal, context-sensitive designs and examine institutional levers (teacher development, assessment policy, and course governance) that sustain these learning-centered features.

## 5 Limitations and future research

This study employed a cross-sectional design, which precludes causal inference, and the stability of profiles and potential transition pathways should be verified through longitudinal tracking. The measures relied primarily on student self-reports and teacher reports, which may be vulnerable to social desirability bias; future studies could incorporate objective fitness assessments and classroom observational coding to strengthen measurement validity. The R3STEP models included only four covariates, so subsequent research should expand this set to include factors such as family support, participation in sports clubs, and teacher self-efficacy. Experimental and quasi-experimental interventions are also needed to test whether targeted strategies can facilitate profile transitions, and to evaluate their cost-effectiveness.

Generalizability is also limited. Participants were drawn from ten universities in Shandong Province, and institutional arrangements, assessment of cultures, and student composition may differ across provinces, university types, and national contexts. Replication in other regions of China and in diverse higher-education settings, alongside tests of measurement invariance and context-specific moderators, is needed to establish the transferability of the profile structure and the proposed instructional prescriptions.

## 6 Conclusion

Synthesizing information criteria and classification quality, the three-class solution was supported on both statistical and educational grounds. Profile membership varied mainly by gender and year, with females more likely to belong to the low-support profile and second-year students more likely to belong to the moderately high profile. These patterns suggest that PE design should prioritize low-threat, self-efficacy-building assessment and instruction for first-year cohorts and female students, supported by tiered goals and progress-referenced formative feedback to encourage upward profile movement. Compared with academic major or urban–rural origin, classroom organization and pedagogy appear to be the most malleable levers, offering a practical route to improve PE learning quality under constrained resources.

## Data Availability

The datasets presented in this study can be found in online repositories. The names of the repository/repositories and accession number(s) can be found below: https://osf.io/evwd7.
